# Unusual case of small bowel intussusception in adult revealing a Peutz-Jeghers syndrome

**DOI:** 10.1259/bjrcr.20210082

**Published:** 2021-11-16

**Authors:** Habib Bellamlih, Ayman El Farouki, Rachid Oulahyane, Nabil Moatassim Billah, Ittimade Nassar

**Affiliations:** 1 Department of Radiology, University Hospital Center IBN SINA, Mohammed V-Souissi University, Rabat, Morocco

## Abstract

Peutz-Jeghers syndrome is a rare genetic disorder characterized by hyperpigmented mucocutaneous macules, hamartomatous polyps of the small intestine, and family history. These hamartomatous polyps can cause intermittent abdominal pain, chronic anemia, or even intussusception. Imaging has an important role in the diagnosis of this syndrome but also in the identification of complications and periodic surveillance. Here, we present a demonstrative case of a Peutz-Jeghers syndrome associated with intussusception in a 16-year-old patient.

## Clinical presentation

A 16-year-old female was admitted into the emergency with acute onset of abdominal pain and vomiting of 04 days. She had a history of recurrent slight abdominal pain for approximately 05 months. Otherwise, there was no history of hematemesis or melena and no significant family history.

On admission, her vital findings were within normal limits. However, physical examination showed slight distension and generalized tenderness in the abdomen. On the skin examination, there were some hyperpigmented macules on the lips and palms.

## Investigations

Laboratory work-up revealed a leukocytosis (white blood cell 13,000/mm^3^) and a slightly elevated C reactive protein of 20.7 mg dl^−1^.

All others blood tests including electrolytes, glucose, serum creatinine, and albumin were within normal limits (serum sodium of 137 mEq/L, potassium 4.2 mEq/L, chloride 106 mEq/L, bicarbonate 19 mEq/L, blood urea nitrogen 8.2 mg dl^−1^, albumin 4.1 g dl^−1^, creatinine 0.6 mg dl^−1^ and glucose of 94 mg dl^−1^).

## Imaging findings

Abdominal ultrasonography demonstrated a characteristic “target sign” in cross-sectional images and a “pseudo kidney sign” in longitudinal images of the periumbilical quadrant. Hence, intussusception was diagnosed according to these findings.

**Figure 1.**
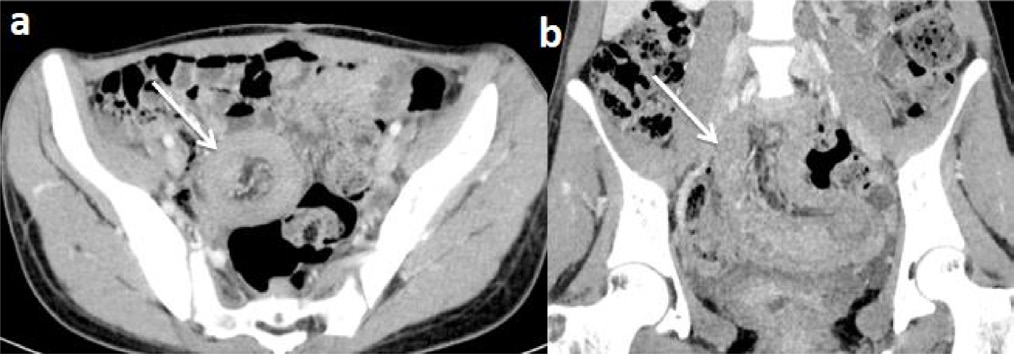
CT scan of the abdomen and pelvis with intravenous contrast on the axial plan (**a**) and coronal reconstruction (**b**) showing ileo-jejunal intussusception containing several polyps (white arrows).

**Figure 2.**
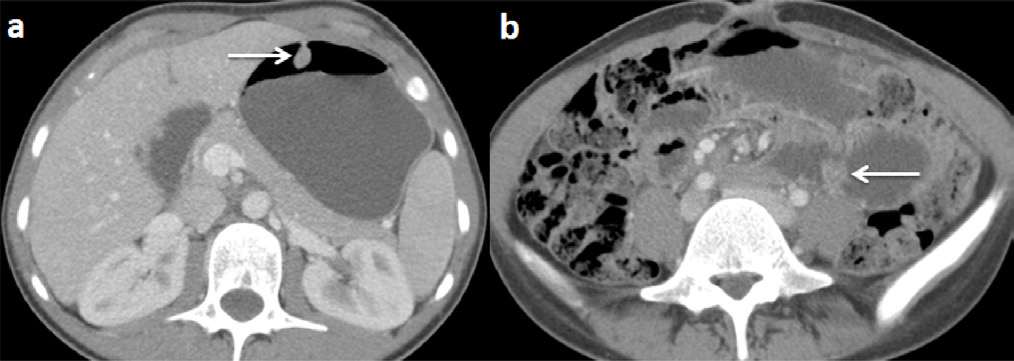
CT scan of the abdomen and pelvis with intravenous contrast on the axial plan showing polyps (white arrows) at the level of the stomach (**a**) and jejunum (**b**).

To further the research etiologic of this intussusception, a CT scan of the abdomen and pelvis with intravenous contrast was performed. It showed ileo-jejunal intussusception containing several polyps (the largest measured 22 mm), with distension of the stomach and proximal small bowel (Figure 1). In addition, there were several others polyps at the level of the stomach, of the second portion of the duodenum, of the jejunum, and the ileum (Figure 2).

Overall and particularly based on the mucocutaneous hyperpigmentation, multiple hamartomatous polyps in the gastrointestinal tract, the diagnosis of the Peutz–Jeghers syndrome (PJS) was made.

## Treatment, outcome, and follow-up

**Figure 3.**
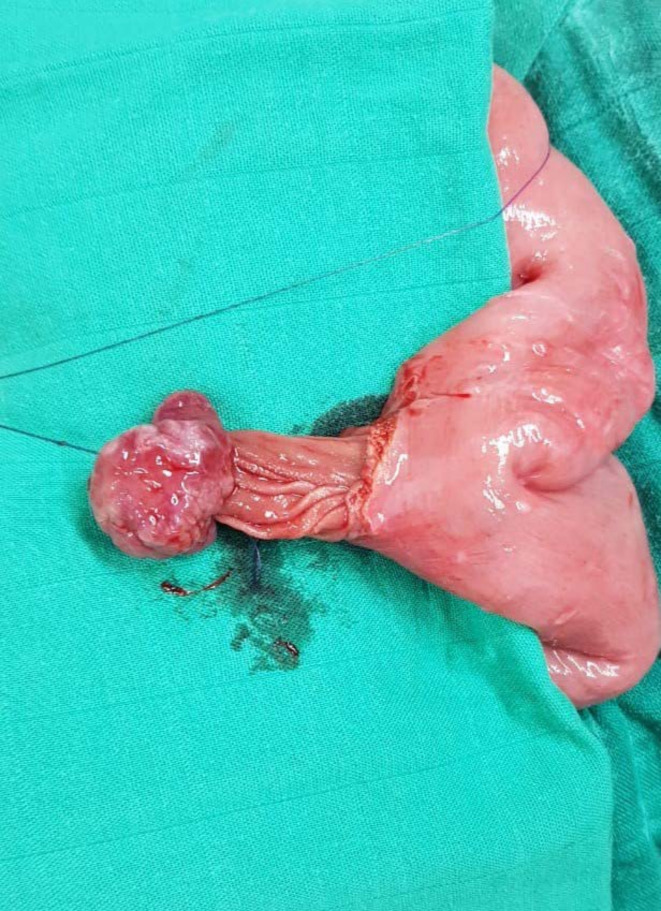
Appearance of ileo-jejunal intussusception after the exploration containing a 3 cm polyp.

**Figure 4.**
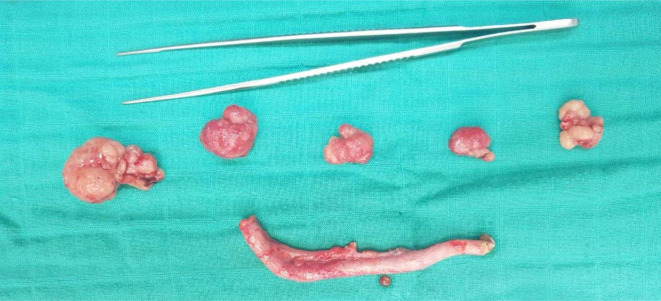
Multiple hamartomatous polyps in the small bowel after resection.

**Figure 5.**
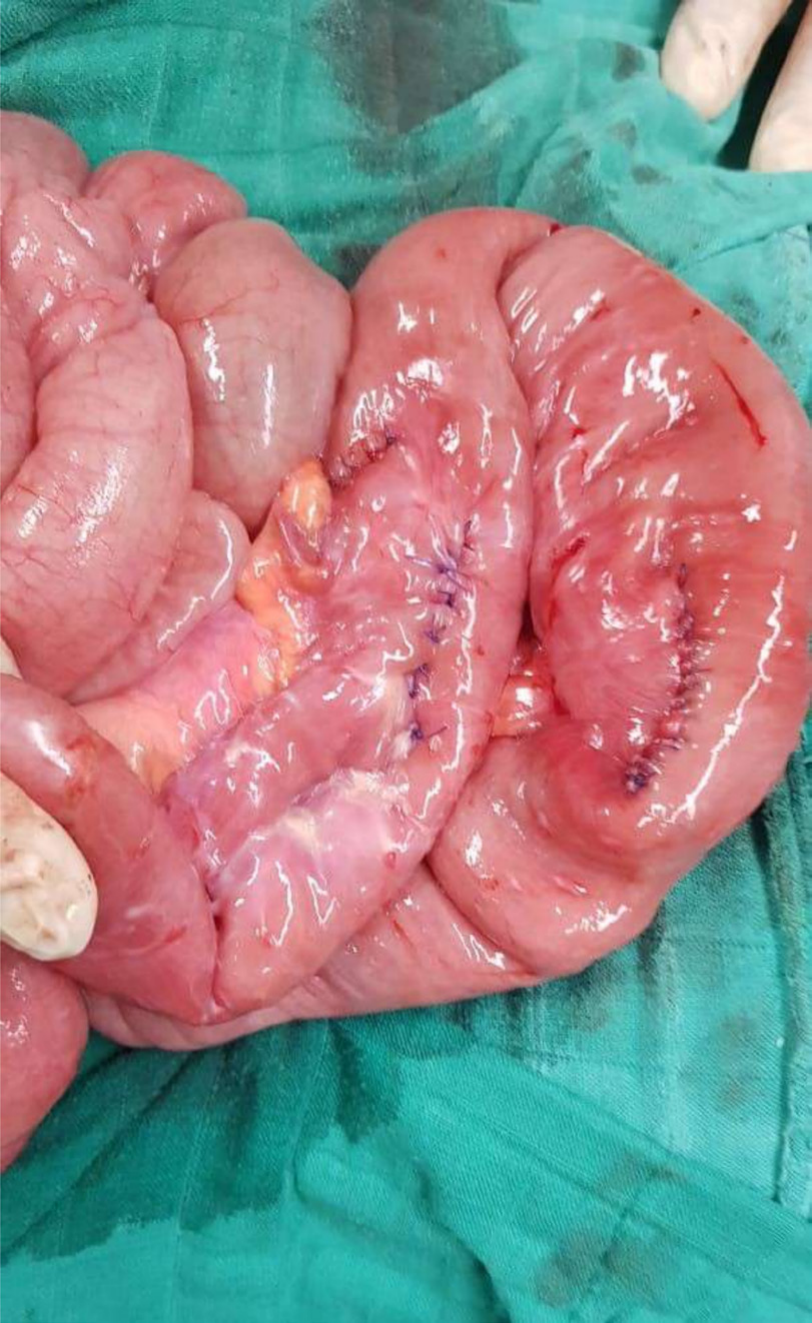
Per-operative view of small bowel after enteric anastomosis.

Emergency laparotomy was decided and confirmed the presence of ileo-jejunal intussusception without areas of necrosis (Figure 3). After a manual reduction, two pedunculated polyps measuring approximately 2 cm and 1.5 cm were revealed. In addition, multiple polyps were palpated throughout the bowel (Figure 4). Moreover, an economic resection of the small bowel carrying away five polyps was performed with side to side-enteric anastomosis (Figure 5) to eradicate any recurrence. The pathological examination of these polyps confirmed their hamartomatous nature and the margins were negative. No sign of malignant transformation was detected.

During the post-operative phase, no event occurred and the patient follows the periodic assessment as recommended.

## Discussion

Intussusception is a relatively common emergency condition in children, and it is usually idiopathic.^1,2^

Adult intussusception, on the other hand, is uncommon, and the etiology identified in 70–90% of patients is most commonly tumor^3,4^; approximately half are benign, and half are malignant.

Intussusception is defined as the condition in which a loop of bowel with its mesenteric “the intussusceptum”, telescopes into the lumen of an adjoining segment “the intussuscipiens’’. This phenomenon impairs peristalsis, obstructs the passage of intestinal content, jeopardizes the mesenteric vascular flow of this portion of the bowel, and can result in intestinal obstruction.^5^

With a reported incidence of 1–5%, intussusception is a rare cause of intestinal obstruction in adults.^6^ In children, in the majority of cases, intestinal intussusceptions are idiopathic whereas, in adults, a demonstrable cause is revealed in 90% of cases.^7^

Pre-operative diagnosis is frequently missed or delayed due to non-specific symptoms in the absence of a pathognomonic clinical picture.

Peutz described PJS in 1921, and Jeghers in 1944 and 1949.^8^ It is a rare condition, with an estimated prevalence of 1 in 100,000 people.^9^

It is caused by autosomal dominant inheritance and is characterized by gastrointestinal hamartomatous polyps, mucocutaneous hyperpigmentation, and multiple neoplasms. This entity can be seen in both male and female patients, and there is no racial predominance.^10^ It is typically diagnosed during the first two decades of life. In most cases (70–80%), a germline mutation in the serine/threonine kinase 11 (STK11/LKB1) tumor suppressor gene on chromosome 19p13.3 has been identified as the cause.^11^

The World Health Organization (WHO) has established the following criteria for diagnosing PJS^11^ clinically:

- Three or more histologically confirmed Peutz–Jeghers polyps,
- Any number of Peutz–Jeghers polyps with a family history of PJS,
- Characteristic mucocutaneous pigmentation with a family history of PJS,
- Any number of Peutz–Jeghers polyps and characteristic mucocutaneous pigmentation.

In our case, the diagnosis was upheld based on the fourth WHO criterion.

Mucocutaneous hyperpigmentation is generally seen on the lips, around the mouth, eyes, nostrils, and on the buccal surface. These hyperpigmented macules are considered hamartomatous in origin without a real potential for malignant transformation.^10^

Although hamartomatous polyps are generally benign, rare occurrences of cancer in these polyps have been observed.^12,13^ Polyps can range in size from 1 mm to 5 cm and can appear in any section of the gastrointestinal tract.

By the age of 20, half of the patients will have suffered intussusception, obstruction, or rectal bleeding, and the median time to the first presentation with polyps is about 11–13 years old.^14,15^

Although there is a link between PJS and intussusception, intussusception is rarely seen as the first sign of PJS.

Our patient was unique in that she developed intussusception as the first complication of PJS when she was 16 years old.

Imaging in PJS is performed for diagnosis, identification of complications, and also for periodic surveillance.^16,17^

Double-contrast gastrointestinal studies performed prior to MRI in an anesthetized child are the most precise method for detecting small bowel polyps. Capsule endoscopy, on the other hand, maybe more useful because it avoids X-ray exposure and the need to sedate the child during an MRI procedure. For uncommon cases, genetic analysis is often indicated.^18,19^

In the case of intussusception associated with PJS, most studies indicate that the CT is the most accurate pre-operative diagnostic tool with a diagnostic accuracy of approximately 83%,^20,21^ which explains our approach while dealing with the diagnosis of our patient.

PJS patients are predisposed to a variety of complications and malignancies, particularly gastrointestinal and breast cancers.^22^ As a result, patients must be monitored regularly to avoid complications and improve their outcomes. The removal of larger polyps and periodic surveillance are intended to reduce the occurrence of complications in PJS. Therefore, according to the American College of Gastroenterology Clinical Guidelines, patients should have an annual complete blood cell count, as well as an annual physical examination that includes a check of the breasts, abdomen, pelvis, and testes.^23^

Polyp removal is the gold-standard for preventing complications. Conducting polypectomy at the right time can prevent the need for repeated emergency procedures and large small intestinal resections, which can lead to short bowel syndrome. Laparotomy and bowel resection were the standard methods for removing symptomatic gastrointestinal polyps for many decades. Some patients, on the other hand, may require multiple surgical resections, which can result in short gut syndrome. A “clean sweep” is a treatment that combines endoscopy and surgery to treat small intestinal polyps in PJS patients. This surgery aims to help with gastrointestinal symptoms and prevent or delay the necessity for repeated abdominal procedures..^24^ We did not practice this procedure due to the emergency and a lack of intraoperative resources.

## Conclusion

Because PJS is a rare condition, patients frequently go undiagnosed for many years. Some patients may present with acute complications such as intussusception for the first time. Early detection in patients and family members, as well as close cancer surveillance, can result in an excellent prognosis for these people.

## Learning points

- Intussusception may be the first lead for PJS.
- A CT scan is critical in diagnosing these cases.
- Patients with PJS should perform periodic surveillance.
- Polyp removal is the golden therapy to prevent complications in PJS.
